# Preparation of a Z-scheme BiVO_4_/Cu_2_O/PPy heterojunction and studying its CO_2_ reducing properties†

**DOI:** 10.1039/d4ra08130g

**Published:** 2025-04-25

**Authors:** Hengxin Ren, Xinyu Sun, Tong Li, Zhixin Ren, Chaoyu Song, Yuguang Lv, Ying Wang

**Affiliations:** a College of Pharmacy, Jiamusi University Jiamusi 154007 China yuguanglv@163.com; b College of Materials Science and Engineering, Jiamusi University Jiamusi 154007 China; c School of Chemistry and Chemical Engineering, Shanghai Jiao Tong University Shanghai 200000 China

## Abstract

Herein, a new Z-scheme BiVO_4_/Cu_2_O/PPy heterostructure photocatalyst was developed with bismuth nitrate and ammonium vanadate as the precursors and sodium dodecyl benzyl sulfonate as the soft template. Through the spatial confinement effect of the sodium dodecyl benzyl sulfonate soft template, peanut-like BiVO_4_ and the BiVO_4_/Cu_2_O/PPy heterojunction were synthesized. The best performance was observed for BiVO_4_/Cu_2_O/PPy (5%), and its photodegradation rate was 6.57 times higher than that of pure BiVO_4_. The mechanism study showed that a light hole (h^+^), superoxide radical (·O_2_^−^), and hydroxyl radical (·OH) participated in the CO_2_ reduction process, which was different from the pure BiVO_4_ reaction system. Therefore, the proposed approach provides a new method for applying BiVO_4_/Cu_2_O/PPy photocatalysts and developing the same type of heterojunction photocatalyst, and they have effective practical application for environmental remediation.

## Abstract 2

Herein, a new Z-scheme BiVO_4_/Cu_2_O/PPy heterostructure photocatalyst was developed with bismuth nitrate and ammonium vanadate as the precursors and sodium dodecyl benzyl sulfonate as the soft template.

## Graphical abstract

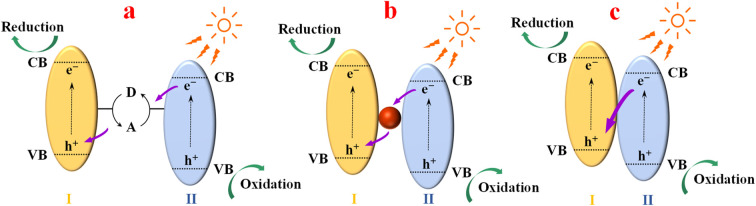

## 1 Introduction

The excessive reliance on fossil fuels is rapidly draining valuable resources, creating an energy crisis, and the greenhouse effect of carbon dioxide that arise from burning of fossil fuels is causing a global climate imbalance.^1^ These factors pose severe challenges to the survival and development of human society. In this context, applying photocatalytic technology enables the conversion of CO_2_ into organic compounds, fuels, and other chemicals of economic value.^2^ This technology demonstrates the potential to directly convert solar energy into chemical energy and helps to reduce the concentration of CO_2_ in the atmosphere while producing valuable chemicals and fuels to achieve a sustainable energy cycle.^3^ However, owing to the high stability of the CO_2_ molecule and its chemical inertness (especially of its sp hybrid carbon, which has a high bond dissociation energy), a higher energy is required to activate and break the CO_2_ molecule.^4^ Hence, developing photocatalysts that provide sufficient activation energy is a critical challenge in the photocatalytic reduction of CO_2_.^5^

**Fig. 1:**
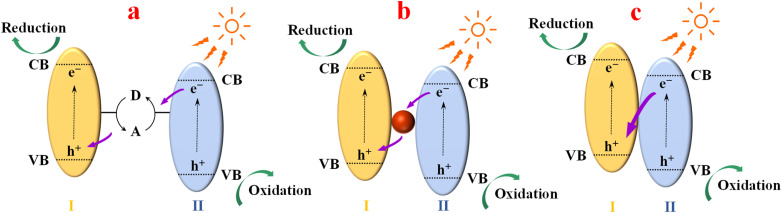
Schematic of the three generations of Z-scheme heterojunction.

**Fig. 2:**
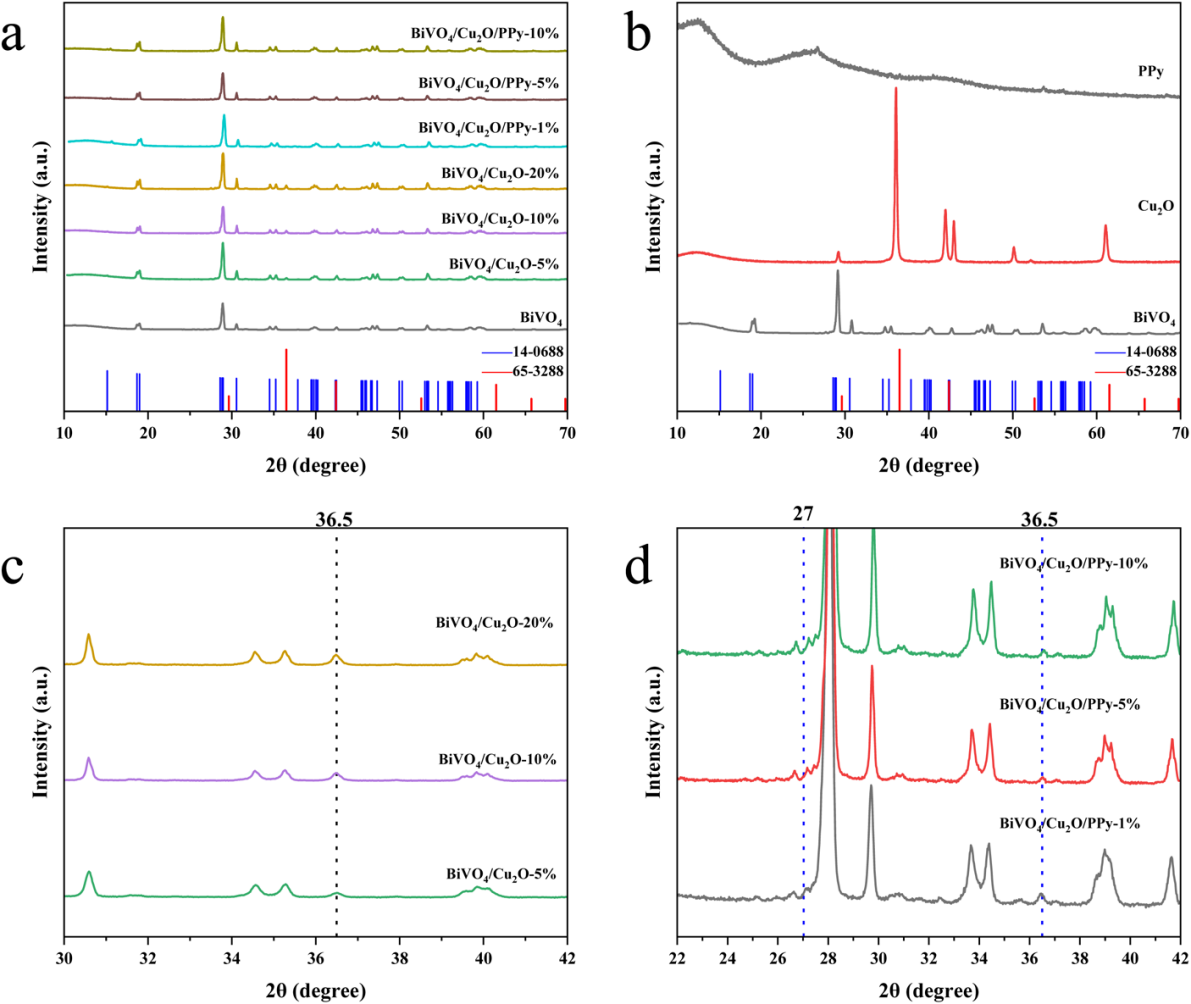
XRD diffraction patterns (a and b) and locally amplified XRD diffraction patterns of the prepared samples (c and d).

**Fig. 3:**
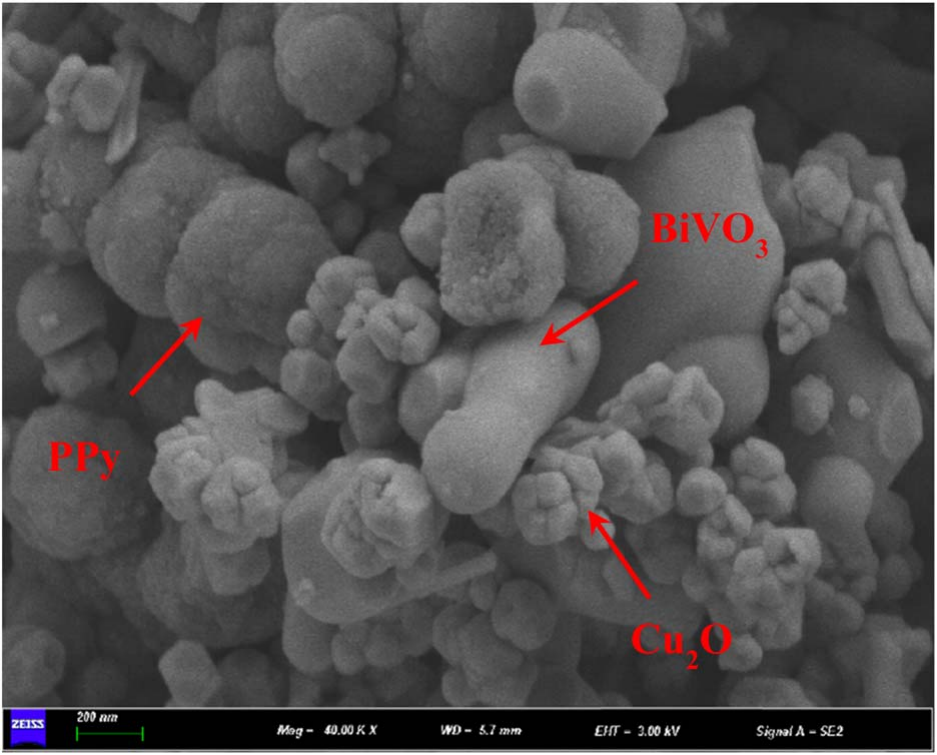
SEM images of BiVO_4_/Cu_2_O/PPy.

Generally, it is difficult for a single component to simultaneously exhibit a wide light absorption range, effective separation of photogenic carriers, abundant reaction sites and strong REDOX capacity. In order to improve photocatalytic efficiency, heterojunctions, which can promote the separation of photogenerated carriers and integrate the respective advantages of each component, are considered one of the most effective ways. According to the development history, there are three generations of Z-scheme heterostructures (Fig. 1):^6–8^ (1) traditional Z-scheme heterojunction with a shuttle REDOX medium (Fig. 1a); (2) all-solid-state Z-scheme heterojunction with an electronic medium (Fig. 1b); (3) direct Z-scheme heterojunction with electron-free media (Fig. 1c). The photogenerated electrons and holes of first-generation heterojunction are consumed by the REDOX medium (A/D), which severely weakens photocatalytic activity. In addition, these REDOX media suffer from the drawbacks of shading, pH sensitivity and suitability for liquid media alone, greatly restricting their wide application. Thus, in order to solve the above-mentioned problems of first-generation heterojunctions, the concept of all-solid Z-scheme heterojunction was proposed. In the all-solid-state Z-scheme heterojunction, an electronic medium with good conductivity binds the two semiconductors tightly, thus replacing the shuttle REDOX medium in the first-generation Z-scheme heterojunction. This structure allows the photogenerated electrons and holes to realize spatial separation, thus improving the photocatalytic activity. However, the cost of introducing electronic media, competitive light absorption and demanding structural control requirements limit its application. As a result, the third generation of direct Z-scheme heterostructures emerged without the need for electronic media. Close contact of A and B semiconductors can produce photogenerated electrons and holes under photoexcitation. Electrons in CB of B can directly combine with holes in VB of A. At the same time, the remaining VB holes in B and the electrons in CB of A maintain the initially strong REDOX capacity.

Cuprous oxide (Cu_2_O) is a reductive semiconductor material with application potential. However, the small *E*_g_ of Cu_2_O (about 2.1 eV) limits the separation efficiency of photogenerated electron–hole pairs, and Cu_2_O is easily disproportionated to form Cu and CuO during the photocatalytic reaction, thus reducing its lifetime and activity.^9^ Bismuth vanadate (BiVO_4_) is a widely studied oxide semiconductor material. As a typical low-cost N-type semiconductor, monoclinic crystal BiVO_4_ has a unique band edge position suitable for water decomposition, with a long hole diffusion distance (about 70 nm), high carrier lifetime (40 ns), and relatively stable photochemical properties.^10^ Therefore, it has significant potential in solar energy conversion and environmental purification. However, the high recombination rate of photoinduced carriers, together with the low carrier mobility and reduction potential limit the photocatalytic activity of BiVO_4_.^11–13^

Combining the high oxidation potential valence band of BiVO_4_ with the high reduction potential conduction band of Cu_2_O to construct the BiVO_4_/Cu_2_O heterojunction can effectively separate the electron–hole pair, improve the interface charge transfer efficiency and enhance the photocatalytic effect.^14^ Kim *et al.*^15^ demonstrated that Z-scheme heterostructures have strong reduction and oxidation potentials by constructing a Z-scheme charge flow on a three-dimensional nanowire array structure (BVO/C/Cu_2_O). The photocatalytic conversion rate of CO_2_ to CO from BVO/C/Cu_2_O nanowire arrays is 3.01 mol g^−1^ h^−1^, which is 9.4 times and 4.7 times that of Cu_2_O mesh and Cu_2_O nanowire arrays, respectively.

Generally, a good electronic medium is needed to reduce the possibility of electron recombination and accelerate the process of electron migration from one semiconductor to another to facilitate charge transfer between heterojunction interfaces. Conjugated polymers such as polyaniline (PANI), polypyrrole (PPy), and polythiophene (PT) are considered to be excellent electrical conductors based on their electrochemical response rather than their structure. PPy is a promising material with magnetic-, optical-, and electronic properties associated with metals or semiconductors. It retains the structure and properties of the polymer, such as ease of processing, flexibility, low toxicity, and adjustable electrical conductivity.^16–19^ However, its poor mechanical properties limit its general application. Many researchers have been working to improve its performance. The doping strategy significantly increases the conductivity and is an effective scheme to promote charge transfer and separation.^20^ Therefore, PPy often acts as a conductive matrix to enhance the composites' electrical conductivity and photocatalytic activity.^21^ In addition, introducing PPy can enhance the dispersion and stability of the composite material in the solution, making it easier to contact the reactants, thus improving the reaction rate and efficiency.^22^

In this study, the high reduction potential of Cu_2_O, high oxidation potential of BiVO_4_ and high conductivity of PPy were combined to construct the Z-scheme photocatalytic heterojunction. The photogenic carrier transfer between Cu_2_O and BiVO_4_ is enhanced in the presence of PPy as an electronic medium, with the reducing capacity of Cu_2_O and the oxidation capacity of BiVO_4_ maintained. In the presence of PPy as an electronic medium, the photogenic carrier transfer between Cu_2_O and BiVO_4_ is enhanced, and the reducing capacity of Cu_2_O and the oxidation capacity of BiVO_4_ are maintained, which realizes the efficient photocatalytic reduction of CO_2_.

## 2 Experiments

### 2.1 Chemicals and reagents

Bismuth nitrate (Bi(NO_3_)_3_·5H_2_O), sodium dodecyl benzene sulfonate (SDBS), nitric acid, sodium hydroxide, ammonium metavanadate (NH_4_VO_3_), anhydrous ethanol, CuSO_4_·5H_2_O, sodium potassium tartrate (NaKC_4_H_4_O_6_·4H_2_O), ammonium sulfate (APS) and sodium hydroxide (NaOH) were purchased from Aladdin Reagent Co., Ltd (Shanghai, China). All the chemicals were analytically pure without further purification. Deionized water was generated in the laboratory.

### 2.2 Preparation of peanut-shaped BiVO_4_

Bi(NO_3_)_3_·5H_2_O (2.43 g) and 0.25 g of SDBS were added to 20 mL of nitric acid solution (2 mol L^−1^) and stirred for 30 min to obtain solution A. NH_4_VO_3_ (0.58 g) and 0.25 g of SDBS were added into 10 mL of sodium hydroxide solution (4 mol L^−1^) and stirred for 30 min to obtain solution B. Under agitation, solution A was slowly added dropwise to solution B. After stirring for 1 h, an orange suspension was obtained. After the pH was adjusted to 7 with NaOH solution, the mixed solution was ultrasonicated for 30 min, and a uniform precursor solution was obtained. The precursor solution was transferred to a polytetrafluoroethylene lined reactor and heated at 180 °C for 12 h at 4 °C min^−1^. After the heating reaction was complete, the product was naturally cooled to room temperature. The impurities and residual surfactants in the samples were removing by alternative wash steps using deionized water and anhydrous ethanol. Finally, BiVO_4_ powder was obtained by vacuum drying at 80 °C for 12 h.

### 2.3 Preparation of BiVO_4_/Cu_2_O

Under agitation, 0.07 mol NaOH and 0.035 mol NaKC_4_H_4_O_6_·4H_2_O were dissolved in 50 mL of distilled water to obtain solution C. A certain amount of BiVO_4_ powder prepared by the method described in Section 2.2 was dissolved in 50 mL of distilled water. Then, CuSO_4_·5H_2_O with different mass fractions were added to the above solution under agitation to obtain solution D. Solution C was added to solution D under continuous magnetic agitation. Subsequently, 50 mL of 0.7 mol L^−1^ ascorbic acid was added to the mixture. After the pH was adjusted to 10, the mixture was centrifuged to obtain a clay yellow precipitate. Impurities in the sediment are removed by performing alternate wash steps using distilled water and anhydrous ethanol several times. The BiVO_4_/Cu_2_O composites with different doping ratios of Cu_2_O (5%, 10%, and 20%) were obtained by vacuum-drying the precipitate at 60 °C for 12 h. In addition, the above steps were repeated without adding BiVO_4_ to prepare Cu_2_O powder.

### 2.4 Preparation of BiVO_4_/Cu_2_O/PPy

**Scheme 1:**
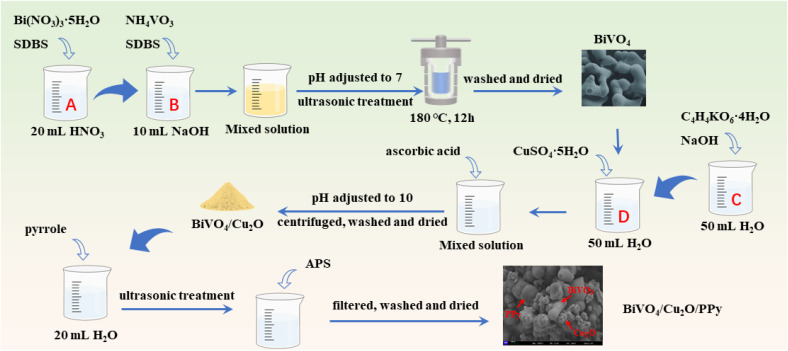
Illustration of the preparation process of the samples.

The pyrrole monomers with different mass fractions were dissolved in 20 mL of deionized water. Then, the prepared BiVO_4_/Cu_2_O complex was added to the pyrrole solution and treated with ultrasound (750 W) for 25 min at room temperature. Ten mL of APS solution (as an oxidizing agent) was slowly added to the above mixture (the molar ratio of pyrrole to APS is 1 : 1). After continuous stirring for 12 h, the solid product was separated by filtration. The solids were washed multiple times by alternating between distilled water and anhydrous ethanol to remove unreacted monomer and oxidizer residues. After washing, the product was vacuum dried at 80 °C for 12 h, and BiVO_4_/Cu_2_O/PPy complexes with different doping ratios of pyrrole (1%, 5%, and 10%) were obtained. The above steps were repeated without adding BiVO_4_/Cu_2_O to prepare pure PPy powder. The preparation of BiVO_4_, BiVO_4_/Cu_2_O, and BiVO_4_/Cu_2_O/PPy is shown in Scheme 1.

### 2.5 Characterization

The crystal structures and compositions of samples were characterized using a SmartLab SE XRD diffractometer at room temperature and the phase analysis was carried out under Cu Kα1 radiation. The morphology, structure, and particle size of the samples were examined using scanning electron microscopy (SEM, Zeiss Gemini 300), transmission electron microscopy (TEM, JEOL JEM-2100F), and high-resolution TEM (HRTEM, JEOL JEM-2100F). The SEM was equipped with a SmartEDX EDS spectrometer detector. Fourier transform infrared spectroscopy (FT-IR) analysis was performed in the 4000–400 cm^−1^ range using a Nicolet iS20 FTIR spectrometer (Thermo Fisher Scientific), with KBr pellets as the medium. The chemical states of the samples were investigated by XPS on a Kratos Axis ULTRA DLD XPS system, which used a monochromatic Al Kα source (*hν* = 1486.6 eV) to record the core-level spectra. The photoluminescence (PL) study was measured with an Edinburgh FLS980 PL spectrometer. The electron spin resonance spectrum (ESR) technique was carried out using electronic paramagnetic resonance spectroscopy (Bruker EMX plus-6/1). Electrochemical impedance spectroscopy (EIS) was conducted using an electrochemical workstation (Shanghai CH Instruments CHI760E).

### 2.6 Photocatalytic CO_2_ reduction

Photocatalytic CO_2_ reduction was carried out using a Labsolar-6A system of PerfectLight with a 300 W xenon lamp as the light source. First, 30 mg of photocatalyst was uniformly dispersed by ultrasonic treatment and laid on the bottom of the reactor. High-purity CO_2_ was then injected into the reactor filled until the air inside the unit was completely replaced. Then, the light source turned on. During the photocatalytic reaction, a 1.0 mL sample of gas is periodically taken every 1 h (for a total of 4 h) from the reactor by a sampling needle for quantitative analysis by gas chromatography.

### 2.7 Band gap value calculation

The band gap of the sample was calculated using formula (1).1
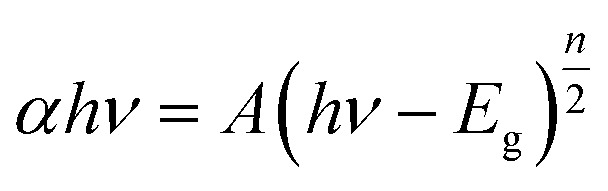
where *α* is the absorption coefficient, *hν* is the photon energy, *A* is a constant equal to 1, and *E*_g_ is the band gap energy. The value of *n* is 4 for indirect bandgap semiconductors and 1 for direct bandgap semiconductors.

The valence band potential and conduction band potential of the sample were calculated using the following empirical formula:

2 *E*_VB_ = *X* + 0.5*E*_g_ − *E*_e_

3 *E*_CB_ = *E*_VB_ − *E*_g_

*E*_CB_ and *E*_VB_ represent conduction band edges and valence band edges, *E*_g_ is the band gap energy, *X* is the absolute electronegativity of the semiconductor, and *E*_e_ is the measurement scale factor of the REDOX level of the reference electrode relative to the absolute vacuum scale (−4.5 eV).

### 2.8 Free radical trapping experiment

A series of free radical trapping experiments were performed to investigate the mechanism of photocatalytic degradation process and identify the active species that play a role in the reaction. Isopropyl alcohol (1 mmol L^−1^), sodium EDTA, potassium dichromate and *p*-benzoquinone were used as scavengers of hydroxyl radical (·OH), hole (h^+^), electron (e^−^) and superoxide anion radical (·O_2_^−^), respectively. The scavenger was mixed with the sample solution in the dark until the adsorption equilibrium was reached. Then, the photocatalytic experiment was carried out, the concentration change of the sample was measured after the reaction was completed, and the corresponding degradation rate was calculated. By comparing the degradation rates of samples with different scavengers, we can infer the active species that play a leading role in the photocatalytic degradation process. This provides key information for the analysis of the photocatalytic mechanism.

## 3 Results and discussion

### 3.1 Phase structure characterization results

The crystallographic properties of the samples were analyzed by X-ray diffraction (XRD). The XRD pattern of BiVO_4_ reveals multiple characteristic diffraction peaks that can be attributed to specific crystal faces of the monoclinic BiVO_4_ system (Fig. 2a and b). The obtained diffraction data are consistent with the powder diffraction file (PDF) card JCPDS 14-0688.^23^ Similarly, the XRD pattern of Cu_2_O corresponds to multiple diffraction peaks of Cu_2_O in the cubic crystal system, matching JCPDS card 65-3288. The high intensity of BiVO_4_ and Cu_2_O diffraction peaks indicates that the samples have excellent crystallinity.

PPy is a typical amorphous polymer. Therefore, sharp diffraction peaks will not be observed in its XRD pattern. A wide reflection at 2*θ* = 27° reflects the presence of PPy.^24^ For composites, a characteristic diffraction peak at 2*θ* = 36.5° indicates successful deposition of Cu_2_O particles on the BiVO_4_ surface (Fig. 2c and d).^25^ However, the diffraction signal of Cu_2_O is relatively weak due to the effective coating of Cu_2_O particles on BiVO_4_ and PPy. In addition, no significant diffraction peaks associated with PPy were detected in the XRD pattern of BiVO_4_/Cu_2_O/PPy composites due to the low doping ratio of PPy and its relatively low diffraction intensity (Fig. 2d). In summary, the loading of Cu_2_O and PPy does not significantly affect the crystal structure of BiVO_4_.

The microstructure and surface morphology of the sample were clearly demonstrated by SEM (Fig. 3). BiVO_4_ exhibits a peanut-like morphology (Fig. S1a†), which is attributed to the regulatory role of SDBS as a “soft template” during crystal growth. The surface of these particles is relatively smooth, and the average diameter is 257.69 ± 69.13 nm (Fig. S1d†). SDBS molecules have a hydrophilic head and a hydrophobic tail. This amphiphilic property causes it to form micellar structures in solution and acts as a structural template to guide the growth of nanomaterials to form nanoparticles with predetermined shapes and sizes. Cu_2_O particles have a regular polyhedral structure with a smooth surface and an average particle size of 74.65 ± 21.68 nm (Fig. S2b and e†). The aggregation of BiVO_4_ and Cu_2_O particles indicates that they are highly crystalline. PPy particles gather irregularly to form a morphology similar to cauliflower, with an average particle size of 151.49 ± 32.59 nm (Fig. S2c and f†). Finally, the Cu_2_O and PPy particles are tightly attached around the BiVO_4_ particles in the BiVO_4_/Cu_2_O/PPy composite (Fig. 3).

**Fig. 4:**
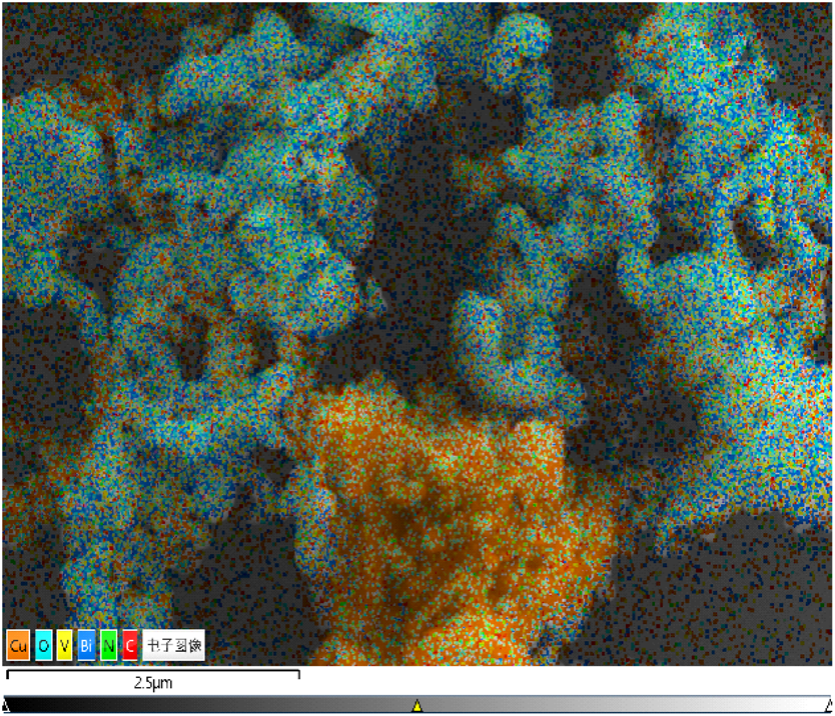
Cumulative EDX elemental mapping signal.

The contents of Bi, V, O, Cu, C and N in BiVO_4_/Cu_2_O/PPy were basically consistent with the actual added ratio according to EDS analysis (Fig. 4, S2 and S3†). The atomic percentage of Bi and V is close to 1 : 1, which is consistent with the atomic composition of BiVO_4_ (Table 1). The element distribution map (Fig. S3†) shows that Bi, V, O, Cu, C and N are uniformly distributed in a specific region, where the distribution of Bi, V and O is consistent with the position of the peanut-like BiVO_4_ particles, and the distribution of Cu and O is consistent with the position of the regular polyhedron structure of Cu_2_O. The peanut-like BiVO_4_ surface is evenly covered with C and N elements, which is attributed to the presence of PPy. SEM and EDS maps confirmed the formation of BiVO_4_/Cu_2_O/PPy composites and the tight binding between them, which is favorable for photocatalytic activity.

**Table 1:** Mass and atomic percentage of each element

Element	Wt (%)	At (%)
C	6.02	22.77
N	0.78	2.52
O	10.49	29.79
V	7.41	6.61
Cu	44.05	31.51
Bi	31.26	6.80

**Fig. 5:**
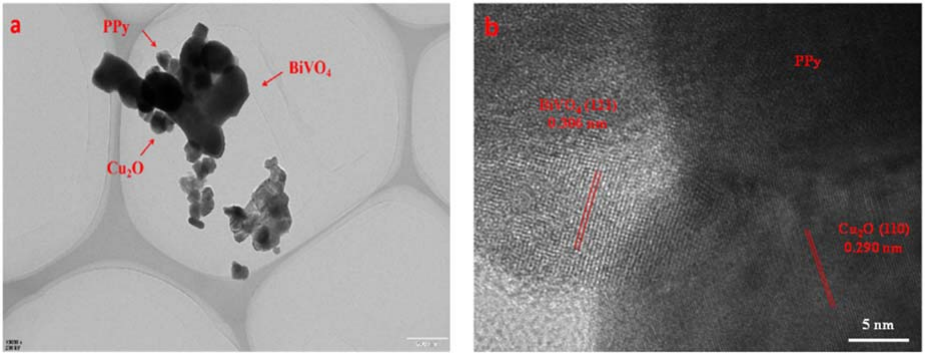
HRTEM images of BiVO_4_/Cu_2_O/PPy nanocomposites: (a) overall coating structure; (b) lattice fringes of BiVO_4_ and Cu_2_O.

The morphological characteristics of BiVO_4_/Cu_2_O/PPy nanocomposites were further verified by HRTEM (Fig. 5). Cu_2_O and PPy surround BiVO_4_, forming a clear coating structure. The measured lattice fringe spacing of 0.306 nm is consistent with the (121) plane of monocline BiVO_4_, while the 0.290 nm interval is consistent with the (110) plane of Cu_2_O (Fig. 5b). These observations suggest that BiVO_4_, Cu_2_O, and PPy form an almost perfect interface contact.

**Fig. 6:**
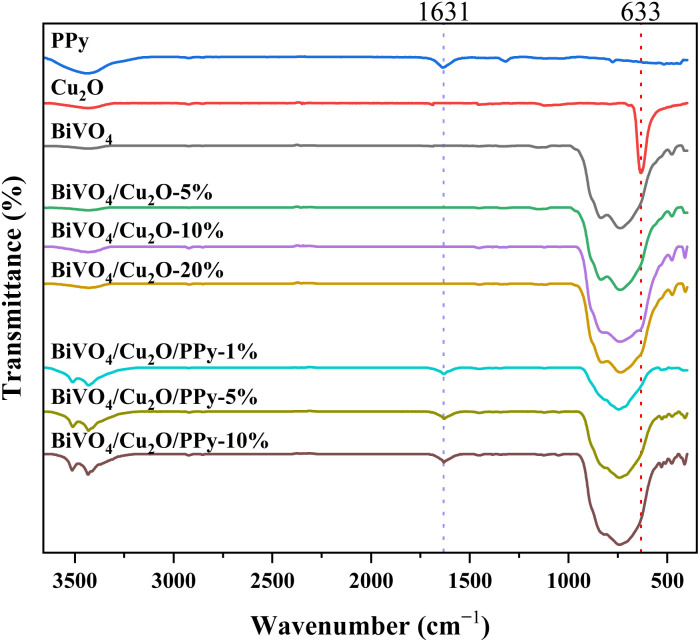
FT-IR spectra of different samples.

The molecular spectral characteristics and chemical group information of the samples were analyzed by FT-IR (Fig. 6). The characteristic peaks at 512 cm^−1^ and 784 cm^−1^ are caused by the stretching vibration of Bi–O and the bending vibration of *ν*_3_ (VO_4_^3−^), and the overlap of these absorption peaks results in a wide peak between 700–900 cm^−1^.^26^ There is a strong absorption peak at 633 cm^−1^, which belongs to the Cu–O bond stretching vibration of the Cu_2_O sample.^27^ The characteristic peaks at 1323 and 1631 cm^−1^ are attributed to the stretching vibrations of –C=N and C=C in the PPy ring.^28^ These results fully proved the successful synthesis of BiVO_4_, Cu_2_O and PPy monomers. In addition, the characteristic peak of Cu_2_O at 633 cm^−1^ overlaps with the wide characteristic peak of BiVO_4_ at 767 cm^−1^, which only causes small changes in the doping ratio of different Cu_2_O samples at 633 cm^−1^, but it proves that the loading of Cu_2_O is successful. The triplet heterojunction samples with different PPy doping ratios showed the characteristic peak of PPy at 1631 cm^−1^, which proved the successful loading of PPy. The stretching vibration of –OH at 3423 cm^−1^ is attributed to the moisture absorbed by the sample.

**Fig. 7:**
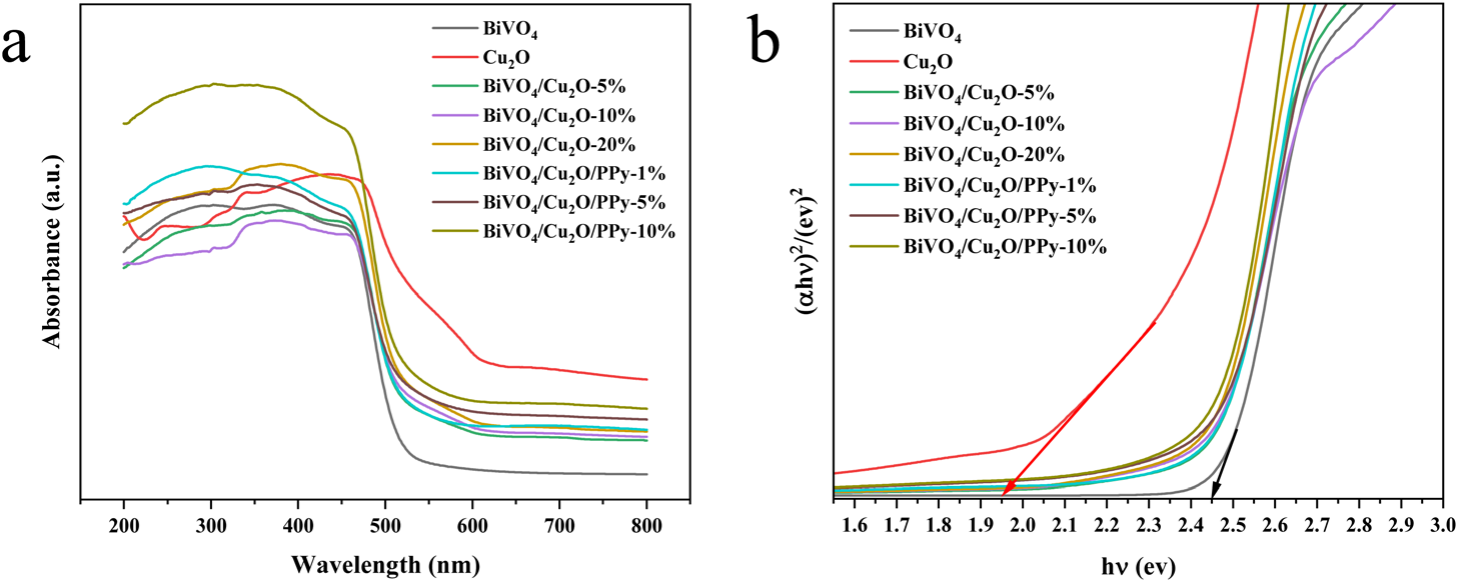
UV-vis diffuse reflectance spectra of different samples (a) and *E*_g_ (b).

The light absorption characteristics of the samples were analyzed by UV-vis DRS. The absorption edges of Cu_2_O and BiVO_4_ are about 730 nm and 525 nm (Fig. 7a), respectively. The absorption edges of the composites are dispersed between 550 nm and 590 nm. All samples have light absorption properties ranging from ultraviolet to visible light, and the visible light absorption of the samples is enhanced after the formation of heterogeneous structures. The visible light absorption capacity of the sample increases with higher doping ratios. BiVO_4_/Cu_2_O recombination ratios of 10% and 5% in BiVO_4_/Cu_2_O/PPy show the highest light absorption intensity compared with similar samples. The utilization efficiency of visible light is improved in the composite samples compared to individual Cu_2_O and BiVO_4_ components, owing to the enhanced absorption properties discussed above.

The bandgap energy of BiVO_4_ and Cu_2_O was estimated using eqn (1), where *α* represents the absorption coefficient, *ν* is the optical frequency, *E*_g_ is the bandgap energy, *A* is a constant, and *n* depends on the transition characteristics of the semiconductor. The value of *n* is 1 for direct transition of BiVO_4_ and Cu_2_O. Thus, the *E*_g_ of BiVO_4_ and Cu_2_O are 2.43 and 1.95 eV (Fig. 7b), respectively. The literature shows that the electronegativity of BiVO_4_ and Cu_2_O are 6.04 (ref. 29) and 4.84 eV,^30^ respectively and the *E*_CB_ and *E*_VB_ values of BiVO_4_ are 0.33 eV and 2.75 eV, respectively, calculated according to formula (2) and (3). The *E*_CB_ and *E*_VB_ values of Cu_2_O are −0.64 eV and 1.31 eV, respectively.

**Fig. 8:**
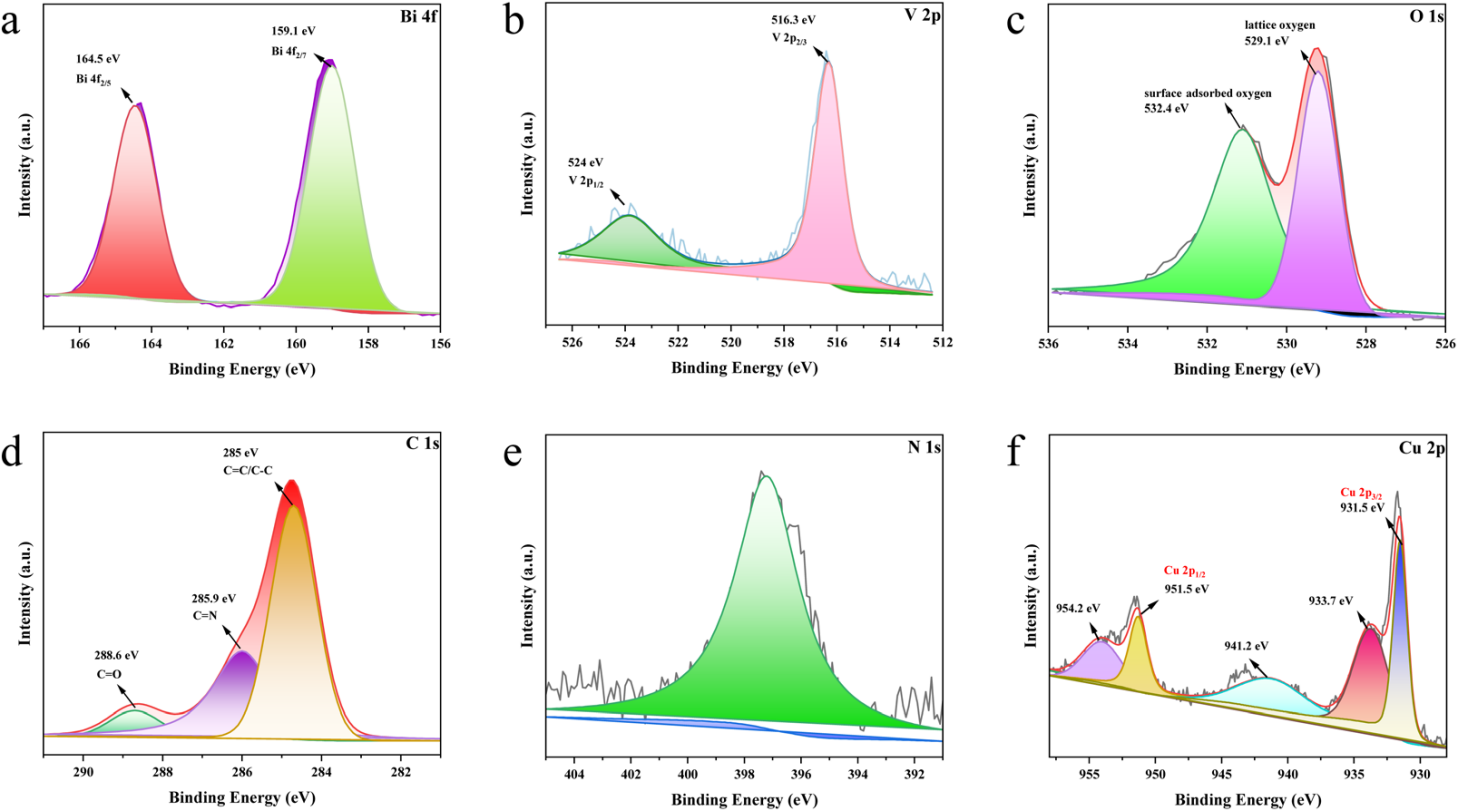
XPS patterns of BiVO_4_/Cu_2_O/PPy samples: Bi 4f (a), V 2p (b), O 1s (c), C 1s (d), N 1s (e), Cu 2p (f).

The XPS spectra of the surface chemical composition and electronic states showed that the samples consisted of Bi, V, O, C, N, and Cu elements (Fig. S4a†), which is consistent with the EDS results. The high-resolution spectrum of Bi 4f can be divided into two characteristic peaks with binding energies of 159.1 eV and 164.5 eV, attributed to Bi 4f_2/7_ and Bi 4f_2/5_, respectively (Fig. 8a), indicating the presence of Bi^3+^ in the composite. The two prominent peaks at 523.6 and 516 eV (Fig. 8b) belong to V 2p_3/2_ and V 2p_1/2_, caused by V^5+^ spin–orbit splitting. The 529.1 and 532.4 eV binding energies in Fig. 8c are attributed to the composite's lattice oxygen and surface-adsorbed oxygen. The chemical states of bismuth, vanadium, and oxygen elements fully demonstrate the presence of BiVO_4_ in the composite sample.^31^ The binding energies of 285 eV, 285.9 eV, and 288.6 eV in Fig. 8e are attributed to C=C/C–C, C–N, and C=O bonds (Fig. 8d). The C=O bond of the C 1s peak demonstrates efficient polypyrrole coating on the BiVO_4_ surface.^32^ In Fig. 8e, the binding energy of 397.2 eV was attributed to the polypyrrole N–H bond. The chemical states of the element demonstrated the presence of PPy.^33^Fig. 8f shows the high-resolution XPS of Cu 2p. Two distinct Cu_2_O signal peaks at 951.5 eV and 931.50 eV correspond to Cu^+^ 2p_1/2_ and Cu 2p_3/2_, respectively. The signal peaks attributed to Cu^2+^ were located at 954.2 eV, 941.2 eV, and 933 eV. The presence of these satellite peaks implies the presence of CuO impurities in the composite photocatalyst, which may be caused by the oxidation of Cu_2_O by oxygen in the air.^34^ The percentage of Bi and V atoms is close to 1 : 1 (Fig. S4b†), which conforms to the composition of BiVO_4_ atoms. The XRD, FT-IR, and XPS tests show that BiVO_4_, Cu_2_O, and PPy were successfully loaded in the composite, which agrees with the morphology characterization results of SEM and TEM.

### 3.2 Photocatalytic properties

**Fig. 9:**
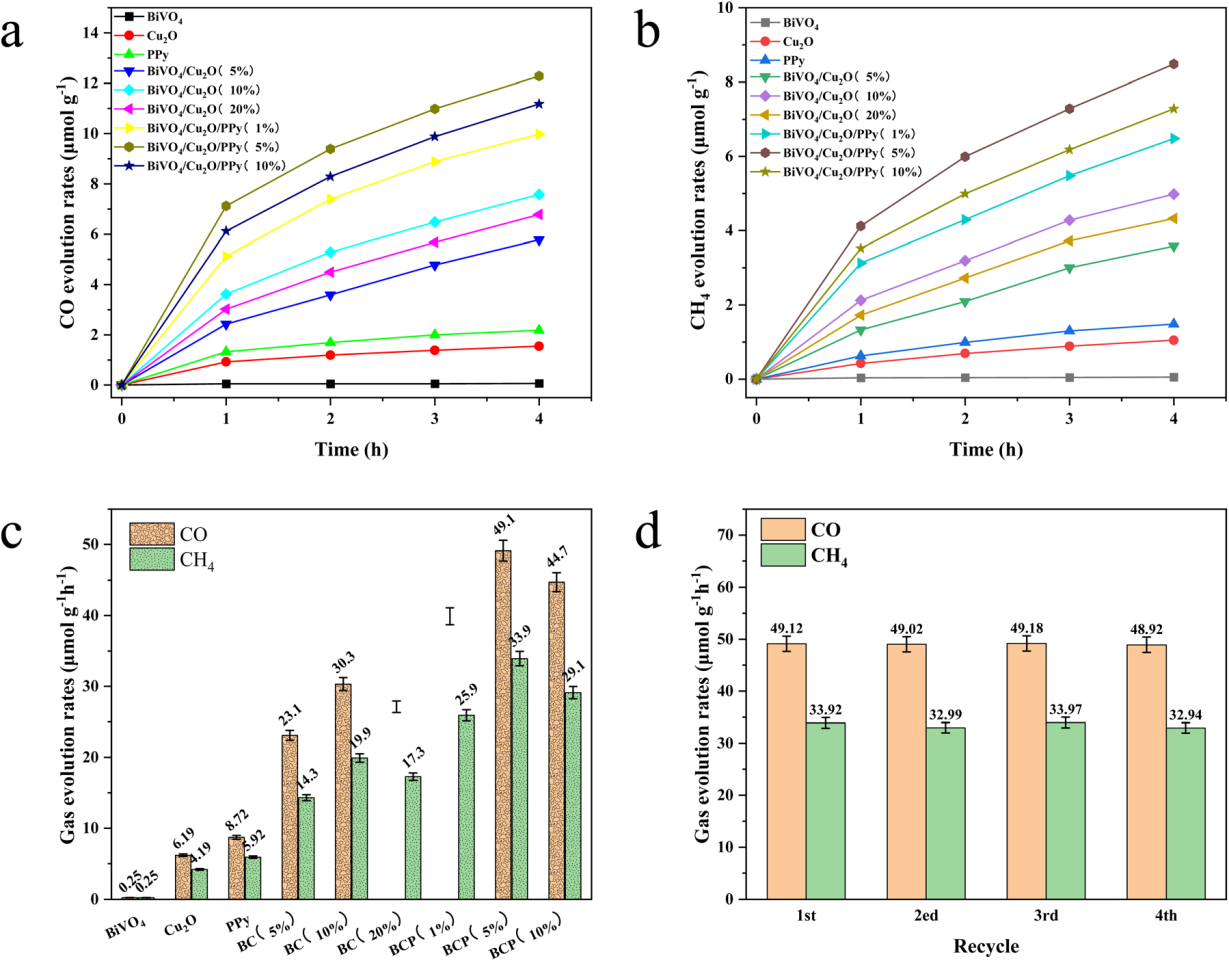
Photocatalytic curves of CO_2_ reduction to (a) CO and (b) CH_4_; bar chart for reduction product generation rate of CO_2_ for different catalysts (c); and stability test of BiVO_4_/Cu_2_O/PPy (5%) (d).

Fig. 9 shows the effect of different catalysts on the photocatalytic reduction of CO_2_ to CO and methane over a total irradiation time of 4 h. BiVO_4_ alone showed poor CO_2_ reduction performance, while Cu_2_O and PPy alone showed relatively good CO_2_ reduction performance (Fig. 9a–c). The yield of CO and CH_4_ produced by Cu_2_O is 24.7 and 16.7 times that of BiVO_4_ samples, respectively. The yield of PPy to CO and CH_4_ was 34.8 and 23.6 times that of BiVO_4_ samples, respectively. The lower reduction potential of BiVO_4_'s conduction band is the main factor leading to its poor CO_2_ reduction. The performance of CO_2_ reduction is significantly improved by the combination of Cu_2_O and BiVO_4_, and is positively correlated with the doping rate of Cu_2_O. The yield of CO and CH_4_ produced by BiVO_4_/Cu_2_O (10%) is 4.89 and 4.74 times higher than that of Cu_2_O samples, respectively. This is due to the fact that the BiVO_4_/Cu_2_O composite has a wider light response range and higher quantum yield than the monomers. Interestingly, the performance of CO_2_ reduction was further improved when PPy was combined with BiVO_4_/Cu_2_O, and is positively correlated with the doping rate of PPy. The CO and CH_4_ yields of BiVO_4_/Cu_2_O/PPy (5%) were 1.62 and 1.71 times higher than those of BiVO_4_/Cu_2_O (10%) samples, respectively. These results indicate that PPy effectively promotes e^−^ transfer as an electronic medium. Moreover, the photocatalytic CO_2_ reduction performance remained good after four cycles of experiments (Fig. 9d). The kinetic equation was obtained by fitting the rate of CO_2_ reduction of all catalysts (Table S1†). Among them, BiVO_4_/Cu_2_O/PPy (5%) shows the largest slope value, indicating that it has the best photocatalytic conversion efficiency.

### 3.3 Photocatalytic mechanism

**Fig. 10:**
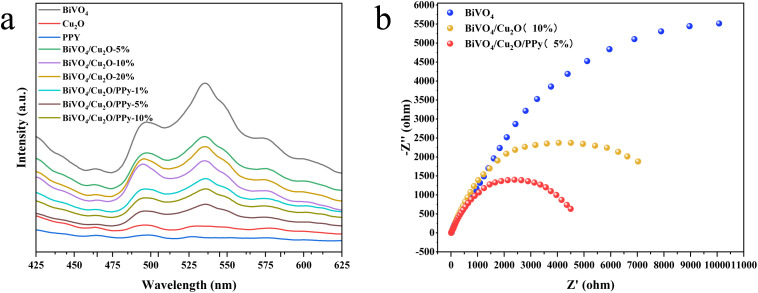
PL spectrum (excitation wavelength: 360 nm) (a) and EIS curve (b) of various photocatalysts.

The mechanism of CO_2_ reduction by the photocatalyst was determined by steady-state PL spectroscopy and an electrochemical impedance test. In the PL diagram (Fig. 10a), the pure BiVO_4_ exhibited the strongest fluorescence spectral absorption peak, which was higher than Cu_2_O and PPy, indicating that its quantum yield is higher and the photogenerated carrier recombination rate is the highest. BiVO_4_/Cu_2_O/PPy has the weakest absorption strength among all samples, indicating that its average carrier lifetime is longer than that of homomonomers and BiVO_4_/Cu_2_O. This indicates that doping with Cu_2_O and PPy can reduce the carrier recombination rate, and the carrier recombination inhibition rate is linearly related to the doping ratio. BiVO_4_/Cu_2_O/PPy (5%) has a small impedance arc curve in the impedance test diagram (Fig. 10b), corresponding to its small resistance and strong electron transport ability. This suggests that doping of Cu_2_O and PPy can promote e^−^ transport. In summary, the separation efficiency and migration rate of photogenerated carriers of BiVO_4_/Cu_2_O/PPy have been significantly improved to obtain excellent photocatalytic CO_2_ reduction performance.

**Fig. 11:**
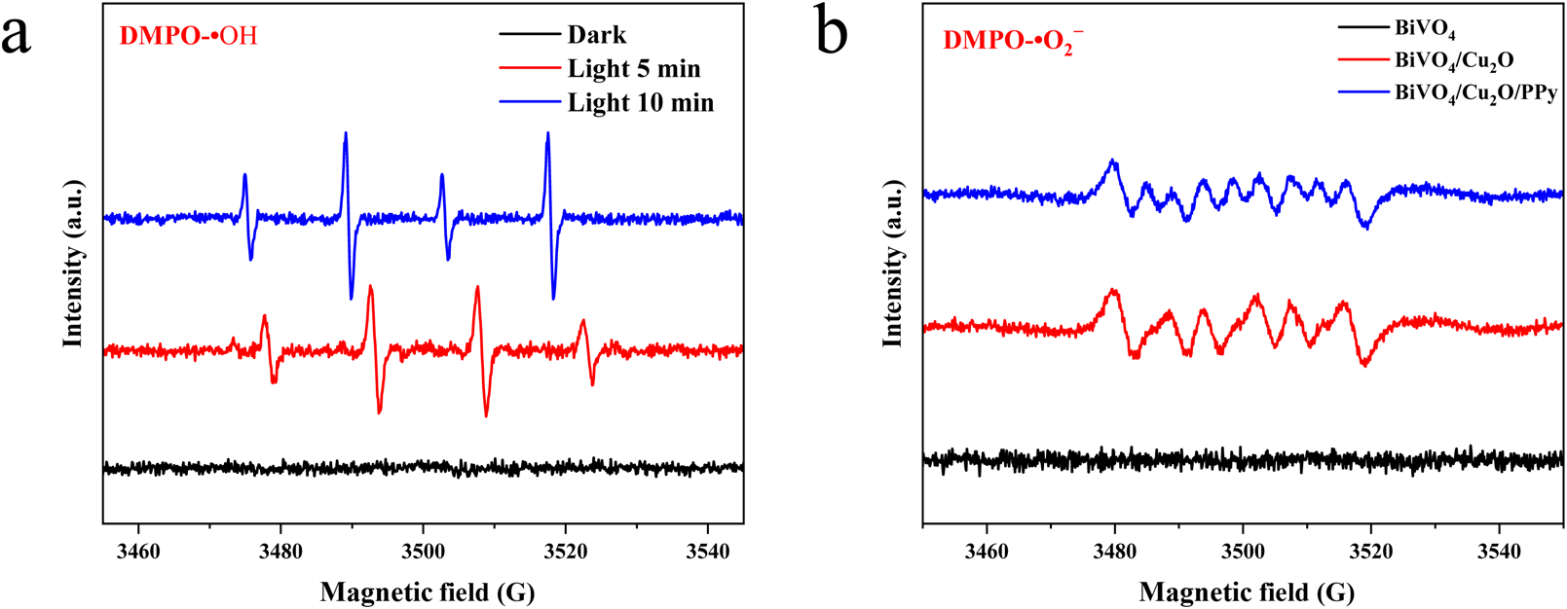
ESR spectra of radical species under varying conditions: (a) DMPO-·OH adducts measured under dark, 5 min light irradiation, and 10 min light irradiation. (b) DMPO-·O_2_^−^ adducts for pristine BiVO_4_, BiVO_4_/Cu_2_O, and BiVO_4_/Cu_2_O/PPy composites.

The REDOX capacity of the sample was measured by ESR. ·OH and ·O_2_^−^ were successfully captured on the surface of BiVO_4_/Cu_2_O and BiVO_4_/Cu_2_O/PPy samples (Fig. 11), indicating that both of these samples have oxidation and reduction capabilities. At the same time, the discovery of ·O_2_^−^ is strong evidence of the Z-scheme electron transport mechanism. However, the simple BiVO_4_ sample does not capture the ·O_2_^−^ because the *E*_CB_ energy level of BiVO_4_ is 0.34 eV, which is lower than the formation potential of ·O_2_^−^ (−0.33 eV) and insufficient to generate ·O_2_^−^. The signals of ·OH and ·O_2_^−^ on the surface of the sample of the ternary composite catalyst are stronger, which indicates that the this ternary heterostructure can more effectively use photogenerated charge for the photocatalytic conversion reaction. These results confirm that the combination of Cu_2_O and PPy can enhance the REDOX capacity of BiVO_4_, thereby improving the efficiency of photocatalytic CO_2_ conversion. These findings have important implications for understanding and designing efficient photocatalysts.

Under visible light, BiVO_4_, Cu_2_O, and PPy produce e^−^ and h^+^ and transfer to the respective CB and VB, respectively. Due to the difference in the Fermi levels of the respective CB and VB, e^−^ will theoretically transfer from PPy to Cu_2_O and then reach the CB of BiVO_4_. Similarly, h^+^ will be transferred from BiVO_4_ to Cu_2_O and then to the LUMO of PPy. The *E*_CB_ potential of BiVO_4_ (0.34 eV) is insufficient to drive ·O_2_^−^ generation (−0.33 eV *vs.* NHE), while the LUMO potential of PPy (2.8 eV *vs.* NHE) also fails to support ·OH production. These thermodynamic limitations contradict the observed radical signals in ESR and trapping experiments, invalidating the proposed electron transport pathway. Consequently, we propose an alternative mechanism.

**Fig. 12:**
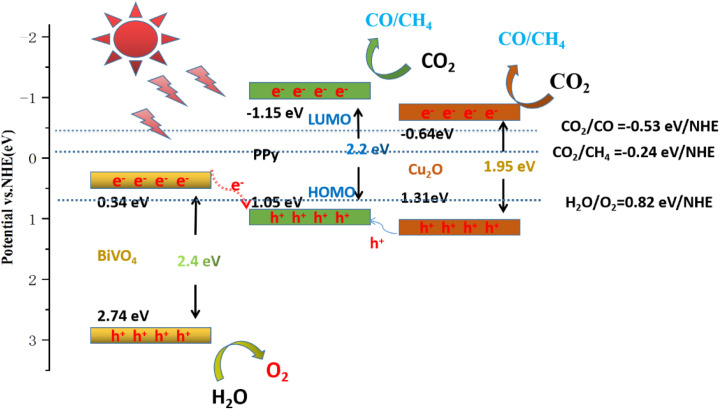
Mechanism of photocatalytic reduction of CO_2_ by BiVO_4_/Cu_2_O/PPy nanocomposites under visible light.

All three photocatalytic materials can be excited by visible light to produce e^−^ and h^+^ (Fig. 12). Subsequently, the photogenic e^−^ on BiVO_4_ (0.34 eV) CB was rapidly transferred to the HOMO of PPy (1.05 eV). However, h^+^ in the Cu_2_O valence band (1.31 eV) can quickly migrate to HOMO and recombine with e^−^. Moreover, photoelectrons on the CB of Cu_2_O would reduce CO_2_, and part of h^+^ on the VB of B of BiVO_4_ would readily bind to generate ·OH, oxidizing H_2_O to produce O_2_. This electron transfer method fits with the Z-scheme heterostructure, and its unique electron transport channel can shorten the migration distance and time of the photogenerated charge, further delaying the reorganization of e^−^ and h^+^, and finally inducing significant photocatalytic activity.^35^

## 4 Conclusion

BiVO_4_ was synthesized using bismuth nitrate as raw material and SDBS as soft template. BiVO_4_/Cu_2_O was obtained by subsequent oxidation. Finally, BiVO_4_/Cu_2_O/PPy was obtained by solution oxidation. XRD, FT-IR, XPS, SEM and TEM results confirm the formation of Z-scheme heterojunction. BiVO_4_/Cu_2_O/PPy (5%) produced 49.1 and 33.9 μmol g^−1^ of CO and CH_4_, respectively, within 4 h, which was 196 and 135 times greater that of BiVO_4_. After four cycles, the CO_2_ reduction rate was maintained. Formation of the Z-scheme charge transfer mechanism shortens the migration distance and time of the photogenerated charge and further delays the recombination of electrons and holes. The photocatalytic performance of the sample was improved. The results provide important theoretical support for the application of BiVO_4_/Cu_2_O/PPy photocatalyst and the design and application of various catalysts in the future.

## Data availability

The authors confirm that the data supporting the findings of this study are available within the article.

## Author contributions

Hengxin Ren: writing – original draft preparation, methodology, formal analysis; Xinyu Sun and Tong Li: writing – original draft preparation, formal analysis, visualization, project administration; Zhixin Ren: writing – review and editing; Yuguang Lv and Ying Wang: data analysis and resources. All authors have read and agreed to the published version of the manuscript.

## Conflicts of interest

The authors declare that they have no known competing financial interests or personal relationships that could have appeared to influence the work reported in this paper.

## Supplementary Material

RA-015-D4RA08130G-s001
