# Supplementary material for: Preparation of a Z-scheme BiVO4/Cu2O/PPy heterojunction and studying its CO2 reducing properties
Source: RSC Adv. 2025 Apr 25;15(17):13313–22. doi: 10.1039/d4ra08130g (PMC12022752; doi:10.1039/d4ra08130g)
Supplement: RA-015-D4RA08130G-s001 [file RA-015-D4RA08130G-s001.pdf]

Supplementary Information (SI) for RSC Advances.
This journal is © The Royal Society of Chemistry 2025

Supplementary information

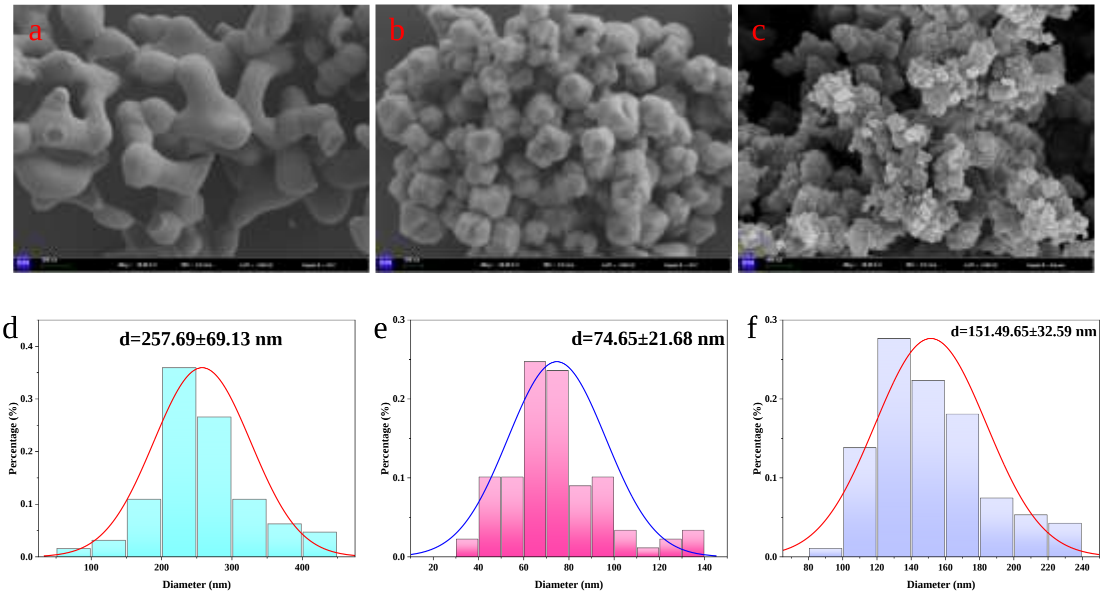

Figure. S1 SEM images of $BiVO_4$ (a), Cu_2O (b) and PPy (c); Particle size distribution of $BiVO_4$ (d), Cu_2O (e) and PPy (f).

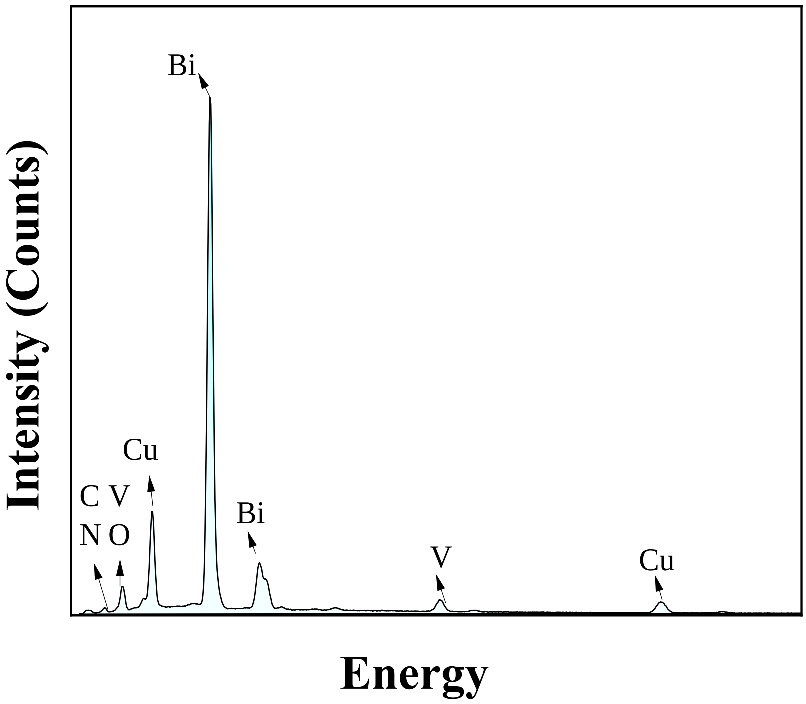

Figure. S2 EDS energy spectrum of $BiVO_4/Cu_2O$/PPy.

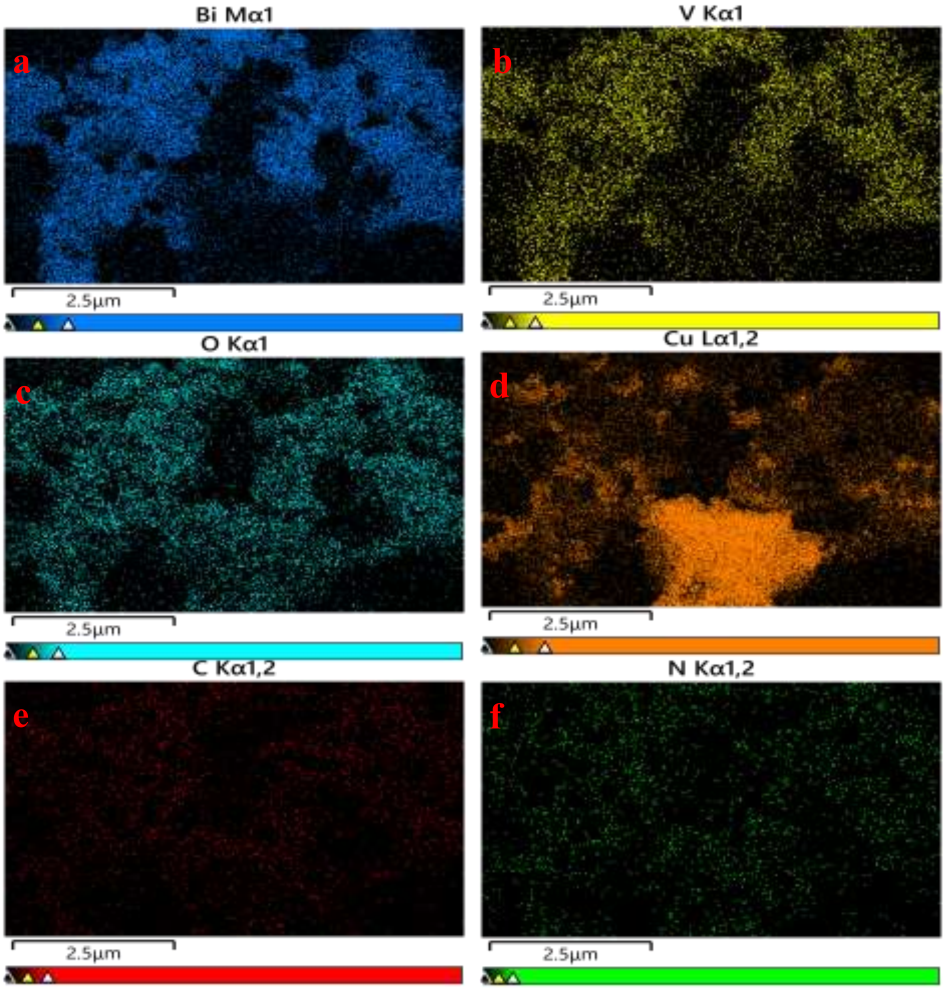

Figure. S3 The corresponding color-coded single-element distribution maps of $BiVO_4/Cu_2O$/PPy containing Bi (a), V (b), O (c), Cu (d), C (e), and N (f).

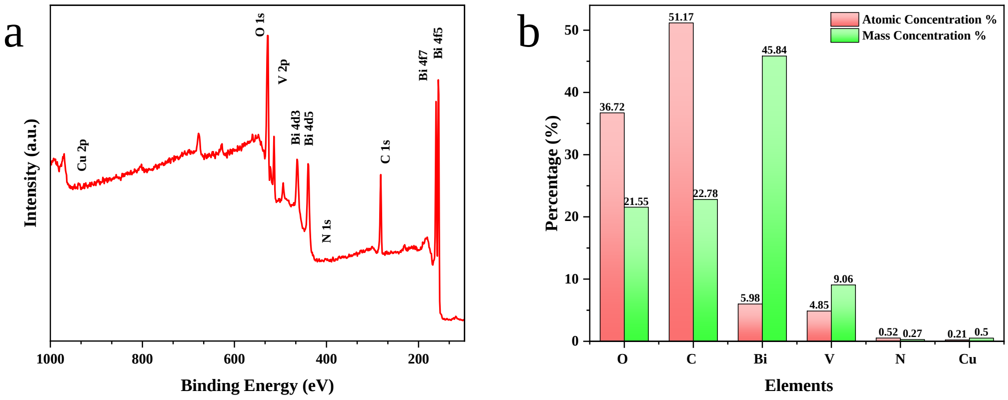

Figure. S4 XPS survey spectrum (a) and Atom (Mass) percent content (b) of $BiVO_4/Cu_2O$/PPy.

Table S1 Kinetic simulation equation of photocatalytic reduction of CO_2 for prepared samples

sample	product	Kinetic fitting parameters			
		Fitting equation	intercept	slope	R^2
$BiVO_4$	CH_4	y=-0.10624x+3.20294	3.20294 ± 0.04721	-0.10624 ± 0.01724	0.94998
	CO	y=0.13047x-3.47465	-3.47465 ± 0.05513	0.13047 ± 0.02013	0.95455
Cu_2O	CH_4	y=0.17001x-0.21003	-0.21003 ± 0.06343	0.17001 ± 0.02316	0.96421
	CO	y=0.19639 x-1.06609	-1.06609 ± 0.14258	0.19639 ± 0.05206	0.94188
PPy	CH_4	y=0.16632x+0.15355	0.15355 ± 0.06781	0.16632 ± 0.02476	0.95755
	CO	y=0.18612x+-0.67228	-0.67228 ± 0.14336	0.18612 ± 0.05235	0.93725
$BiVO_4/Cu_2O$（5%）	CH_4	y=0.17901 x+1.82934	1.82934 ± 0.07268	0.17901 ± 0.02654	0.95789
	CO	y=0.22907x+1.07082	1.07082 ± 0.08146	0.22907 ± 0.02974	0.96997
$BiVO_4/Cu_2O$（10%）	CH_4	y=0.21838 x+1.48375	1.48375 ± 0.11038	0.21838 ± 0.04031	0.93621
	CO	y=0.24339 x+0.93298	0.93298 ± 0.0649	0.24339 ± 0.0237	0.98139
$BiVO_4/Cu_2O$（20%）	CH_4	y=0.19814 x+1.66272	1.66272 ± 0.07905	0.19814 ± 0.02887	0.95928
	CO	y=0.23579 x+1.24307	1.24307 ± 0.09923	0.23579 ± 0.03623	0.95490
$BiVO_4/Cu_2O$/PPy（1%）	CH_4	y=0.24169 x+1.1078	1.1078 ± 0.09923	0.24169 ± 0.03623	0.95698
	CO	y=0.28506 x+0.53072	0.53072 ± 0.11085	0.28506 ± 0.04048	0.96124
$BiVO_4/Cu_2O$/PPy（5%）	CH_4	y=0.28935x+0.6474	0.6474 ± 0.08799	0.28935 ± 0.03213	0.97593
	CO	y=0.33456 x+0.01175	0.01175 ± 0.12248	0.33456 ± 0.04472	0.96550
$BiVO_4/Cu_2O$/PPy（10%）	CH4	y=0.26575 x+0.9006	0.9006 ± 0.0967	0.26575 ± 0.03531	0.96590
	CO	y=0.30713 x+0.31361	0.31361 ± 0.13345	0.30713 ± 0.04873	0.95207
